# Identifying Modifiable System-Level Barriers to Living Donor Kidney Transplantation

**DOI:** 10.1016/j.ekir.2022.08.028

**Published:** 2022-09-09

**Authors:** Shaifali Sandal, Ian Schiller, Nandini Dendukuri, Jorane-Tiana Robert, Khaled Katergi, Ahsan Alam, Marcelo Cantarovich, Julio F. Fiore, Rita S. Suri, David Landsberg, Catherine Weber, Marie-Chantal Fortin

**Affiliations:** 1 Division of Nephrology, Department of Medicine, McGill University Health Centre, Montréal, Québec, Canada; 2 Research Institute of the McGill University Health Centre, Montréal, Québec, Canada; 3 Departments of Medicine and Epidemiology, Biostatistics and Occupational Health, McGill University Health Centre, Montréal, Québec, Canada; 4 Department of Surgery, McGill University Health Centre, Montréal, Québec, Canada; 5 Department of Medicine, University of British Columbia, Vancouver, British Columbia, Canada; 6 Division of Nephrology, Department of Medicine, Centre hospitalier de l’Université de Montréal, Montréal, Québec, Canada; 7 Centre de recherche du Centre hospitalier de l’Université de Montréal, Montréal, Québec, Canada

**Keywords:** barriers, health professionals, health system, living donor kidney transplantation

## Abstract

**Introduction:**

Studying existing health systems with variable living donor kidney transplantation (LDKT) performance and understanding factors that drive these differences can inform comprehensive system-level approaches to improve LDKT. We aimed to quantify previously identified barriers and estimate their association with LDKT performance.

**Methods:**

We conducted a cross-sectional survey of health professionals (HPs). Statements, rated on a Likert scale of "strongly disagree” to “strongly agree”, captured themes related to communication; role perception; HP’s education, training and comfort; attitudes; referral process; patient; as well as resources and infrastructure. The percentage who agreed with these statements was analyzed and compared by LDKT performance (living donation rates higher or lower than the national average) and participant characteristics.

**Results:**

We obtained 353 complete responses. Themes related to poor communication, poor role perception, and HPs education or training or comfort emerged as barriers to LDKT. When compared with HPs from high-performing provinces, those from low-performing provinces had lower odds of agreeing that their province promoted LDKT (adjusted odd ratio [aOR] = 0.27, 95% confidence interval [CI]: 0.16–0.48). They also had lower odds of initiating discussions about LDKT (aOR = 0.30, 95% CI: 0.17–0.55), and higher odds of agreeing that the transplant team is best suited to discuss LDKT (aOR = 2.64, 95% CI: 1.60–4.33) and that more resources would increase LDKT discussions (aOR = 2.06, 95% CI: 1.25–3.40). Nonphysician role and less than 10 years of experience were associated with the level of agreement across several themes. Creating guidelines, streamlining evaluations, and improving communication were ranked as priorities to increase LDKT.

**Conclusion:**

There are system-level barriers to LDKT and some were more prevalent in low-performing provinces. Interventions to eliminate them should be implemented in conjunction with patient-level interventions as part of a comprehensive system-level approach to increase LDKT.

## Graphical abstract

**Figure:**
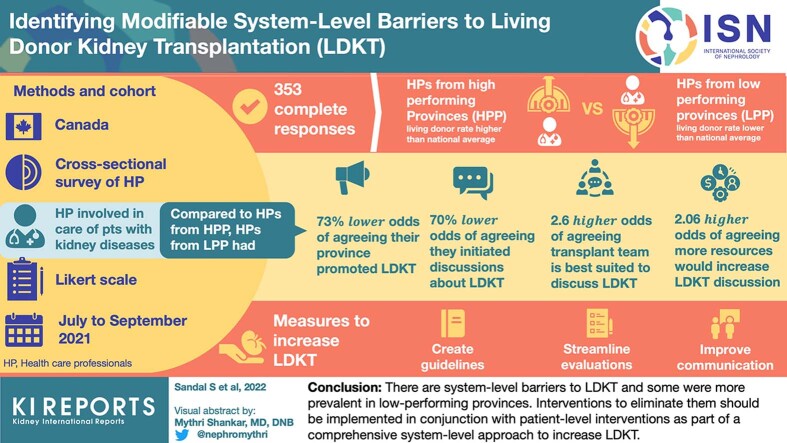

LDKT is the preferred therapeutic option for patients with end-stage kidney disease and is cost-effective for health care systems when compared with dialysis.^1^, ^2^, ^3^, ^4^ Thus, there is global interest in increasing LDKT and improving patient access to this therapy. Nevertheless, current and often unidimensional approaches to increase LDKT have led to modest improvements, and some argue, are contributing to disparities in access to LDKT.^1^^,^^3^^,^^5^, ^6^, ^7^, ^8^, ^9^ Overall, there is growing recognition that efforts to increase LDKT should include a comprehensive system-level approach to understanding and addressing barriers and inefficiencies that patients and their donors face.^1^, ^2^, ^3^^,^^10^, ^11^, ^12^, ^13^, ^14^

Investigating current health systems with variable LDKT rates and understanding factors that drive these differences may provide rich insights into these system-level barriers and inefficiencies. Such investigative work can be pursued in the Canadian context. Canada provides universal health care to its citizens and health care is federally funded. However, the provision of services is under provincial mandates, which has led to real-life differences in LDKT performance.^15^ The national living donor rates averages about 15 donors per million population annually, however, these rates range from 6 to 23 living donors per million population across provinces.^16^, ^17^, ^18^, ^19^ Some provinces have consistently outperformed the national average, while others have had consistently lower rates. A better understanding of what drives these differences may better inform real-life system-level barriers to LDKT.

Obtaining patient perspectives on system-level barriers to LDKT is an important approach that many have explored.^14^^,^^20^ However, HPs are also an integral part of health systems. They are known to play an important role in improving access and quality of care delivered to patients,^8^^,^^21^ and may provide unique insights into system-level inefficiencies that impede LDKT. We previously conducted an interpretive descriptive study of HPs from 3 Canadian provinces with variable LDKT performance.^22^ Indeed, we identified several themes that were perceived as barriers to LDKT***.***^22^ These pertained to communication, role perception, referral process, HP’s education or training or comfort, attitudes, patient as the barrier, and resources and infrastructure. HP characteristics may have influenced the perception of these barriers; however, this remains to be explored. Also, quantifying these barriers on a larger scale and determining their association with LDKT performance can pragmatically inform system-level barriers to LDKT and interventions that have real-world application.

Thus, we aimed to quantify system-level barriers to LDKT that we have previously identified in our qualitative work and estimate their association with LDKT performance. We also aimed to determine if HP’s characteristics influenced responses and what HPs thought should be priorities to increase LDKT.

## Methods

This is a cross-sectional survey (supplement [Survey]) of HPs involved in the care of patients with kidney failure or those who facilitate transplantation. The survey was conducted from July 15 to September 14, 2021, in English and French. The study was approved by the Research Ethics Board of the McGill University Health Centre.

### Setting

As of 2020, the population of Canada was 38 million people, and the population density was fairly varied with 75% of the population residing in the provinces of British Columbia, Ontario, and Québec.^23^ As of December 2020, over 3050 patients were waiting for a kidney alone transplantation.^16^ Prior to the disruptions caused by the pandemic, most kidney transplantations performed in Canada were from deceased donors. For example, in 2019, a little over 1700 kidney alone transplantations were performed, of which only 534 were from living related or unrelated donors.^24^ In 2020, there was a decline in transplantations due to the COVID-19 pandemic. As mentioned above, there are real-life differences in LDKT performance. We defined high-performing provinces as those with living donor rates consistently above the national average of 15 living donors per million population over the past decade (British Columbia, Ontario, Alberta, Manitoba, and North-West Territories) whereas low-performing provinces were those whose rates were consistently below the national average (Québec, Nova Scotia, New Brunswick, Saskatchewan, Newfoundland and Labrador, and Prince Edward Island).^16^, ^17^, ^18^, ^19^

### Survey Design

For methodological guidance on survey creation, we followed the works of Boynton, Gillham, and Oppenheim.^25^, ^26^, ^27^ For content, we developed statements to capture themes identified in our previous work (Column 1 of Table 1).^22^ Briefly, this entailed thematic analysis to analyze the data stemming from the interviews with HPs involved in transplant co-ordination. To minimize social acceptability bias, we formulated the statements to be as neutral as possible and periodically alternated positive and negative verbal anchors.^28^ To reduce the risk of acquiescence bias, Likert scales were used.^29^ The initial survey (drafted by SS) was circulated among the study team and submitted to several rounds of revisions to refine the statements. Subsequently, the survey was pilot-tested among 16 English or French-speaking HPs (prior to the COVID-19 pandemic) and revised according to their feedback. The pilot responses were discarded but the testers were contacted again for participation.

**Table 1:** Characteristics of the participants

Characteristic	%
Province’s LDKT performance^a^	
High	64.9
Low	35.1
Role	
Physician	29.2
Nephrologist (donor)	2.6
Nephrologist (referring)	18.7
Nephrologist (transplant)	6.5
Trainee doctor	1.4
Nonphysician	70.8
Administrative assistant	2.3
Dietician	2.8
Nurse (chronic kidney disease)	11.9
Nurse (dialysis)	25.2
Nurse (manager)	7.9
Other	8.8
Patient partner	0.3
Social worker	6.0
Technician (dialysis)	1.1
Transplant coordinator	4.5
Age	
<35	15.0
35–44	24.7
45–54	33.7
55–64	16.7
≥65	4.8
Prefer not to answer	5.1
Self-identified Race	
Caucasian or White	73.9
Other	19.3
Asian	8.8
Black or African American	1.1
Indigenous	0.9
Middle Eastern or North African	0.9
Mixed	1.7
Other	1.1
Pacific Islander	0.3
South Asian	4.5
Prefer not to answer	6.8
Self-identified Gender	
Female	75.6
Male	20.1
Other	0
Prefer not to answer	4.3
Yr of experience	
≤10	39.9
>10	60.1

LDKT, living donor kidney transplantation.

a High-performing provinces were those with living donor rates consistently above the national average of 15 living donors per million population (British Columbia, Ontario, Alberta, Manitoba, North-West Territories) whereas low-performing provinces were those whose rates were consistently below (Québec, Nova Scotia, New Brunswick, Saskatchewan, Newfoundland and Labrador, Prince Edward Island).

### Administration and Recruitment

**Figure 1:**
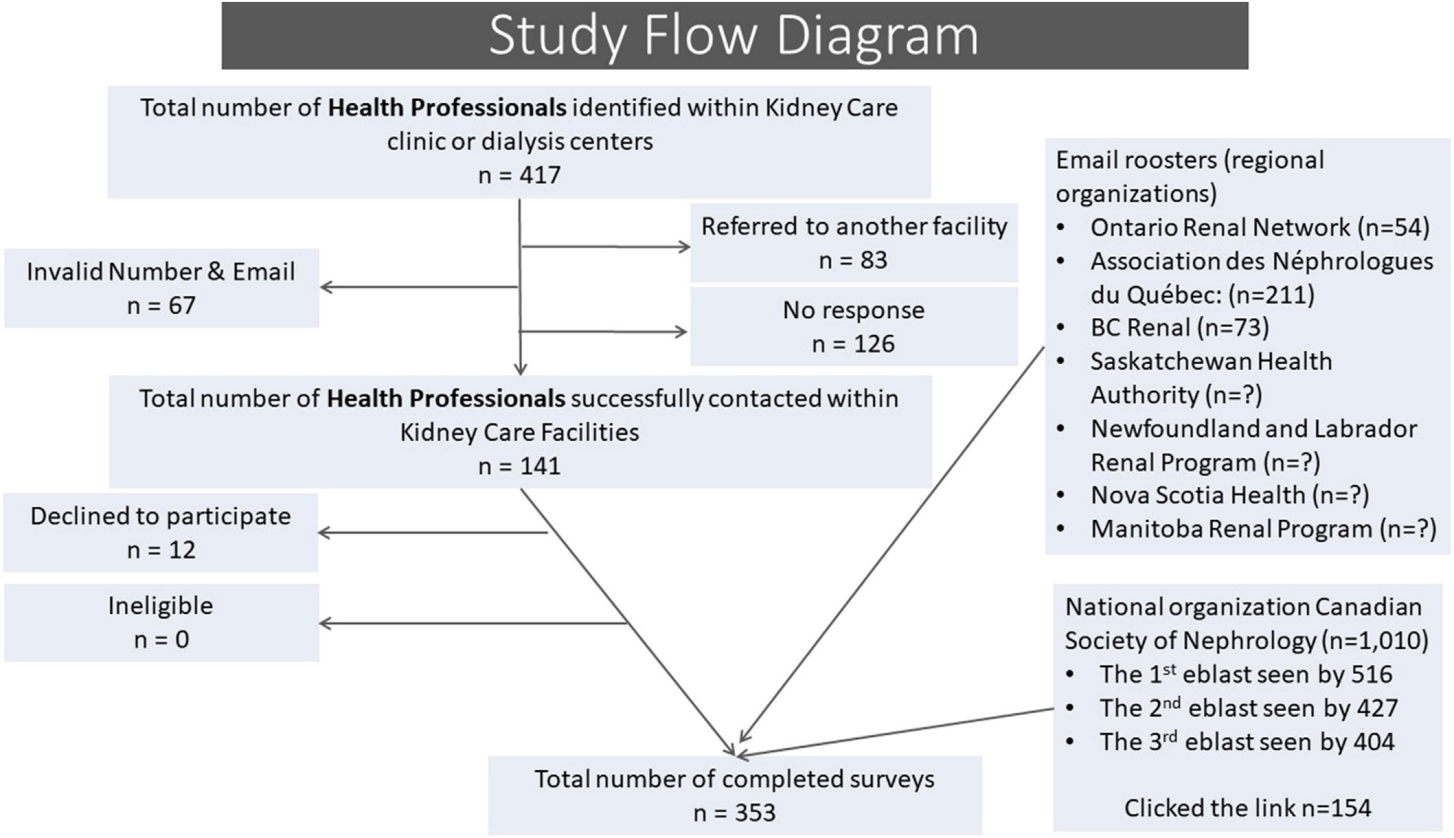
Study flow diagram (some organizations did not share the number of individuals in their email roster)

Links to the survey were sent electronically using the SurveyMonkey platform. The option of paper copy was also offered, but all participants preferred to respond electronically. We used a 2-fold recruitment strategy. First, we contacted local and national professional orders and societies to assist with survey dissemination (see Acknowledgments). Second, to ensure that we also obtained the perspective of community HPs who may not be part of these academic organizations, we used the Google search engine and compiled a list of directors or managers of all dialysis units and nephrology clinics across Canada. Because there was a significant overlap across these strategies, we could not calculate the precise response rate; however, we have summarized our approach and number of individuals contacted in Figure 1.

### Exposure and Outcomes

The main outcome was the level of agreement with each statement rated on a Likert scale. The exposure was provincial LDKT performance and HP characteristics. Given the variability in population density, we ensured adequate representation from each province by calculating the number of responses per million population in each province.^30^ We also sought to analyze if the following 4 well-recognized baseline characteristics of the HP that are known to influence care provision were associated with the level of agreement: the role of participant, years of experience, self-reported gender, and race.^31^, ^32^, ^33^, ^34^, ^35^, ^36^

### Data Analysis

Baseline characteristics were tabulated as category percentages. We calculated the percentage of respondents who agreed or strongly agreed with the statement as rated on a Likert scale of 1 to 5 (strongly disagree–strongly agree). For some questions, the option of not applicable or do not know was allowed (based on feedback from pilot testing) and not included in the analysis. We then conducted a logistic regression of the level of agreement with these statements (agreement vs. disagreement or neutral) by LDKT performance in a univariate and multivariate model. In the latter analysis, we adjusted estimates by the respondent’s self-identified gender, their role in the multidisciplinary team (physician vs. nonphysician), race (White and non-White), and years of experience (>10 years and ≤10 years). We also used this model to study the association of the level of agreement with these statements and the HP’s characteristics. Lastly, we asked HPs to prioritize approaches to increase LDKT and ranked them collectively and by each province. We conducted a descriptive analysis of commentary. All analyses were performed using R statistical software, version 4.0.3 (Vienna, Austria).

## Results

**Figure 2:**
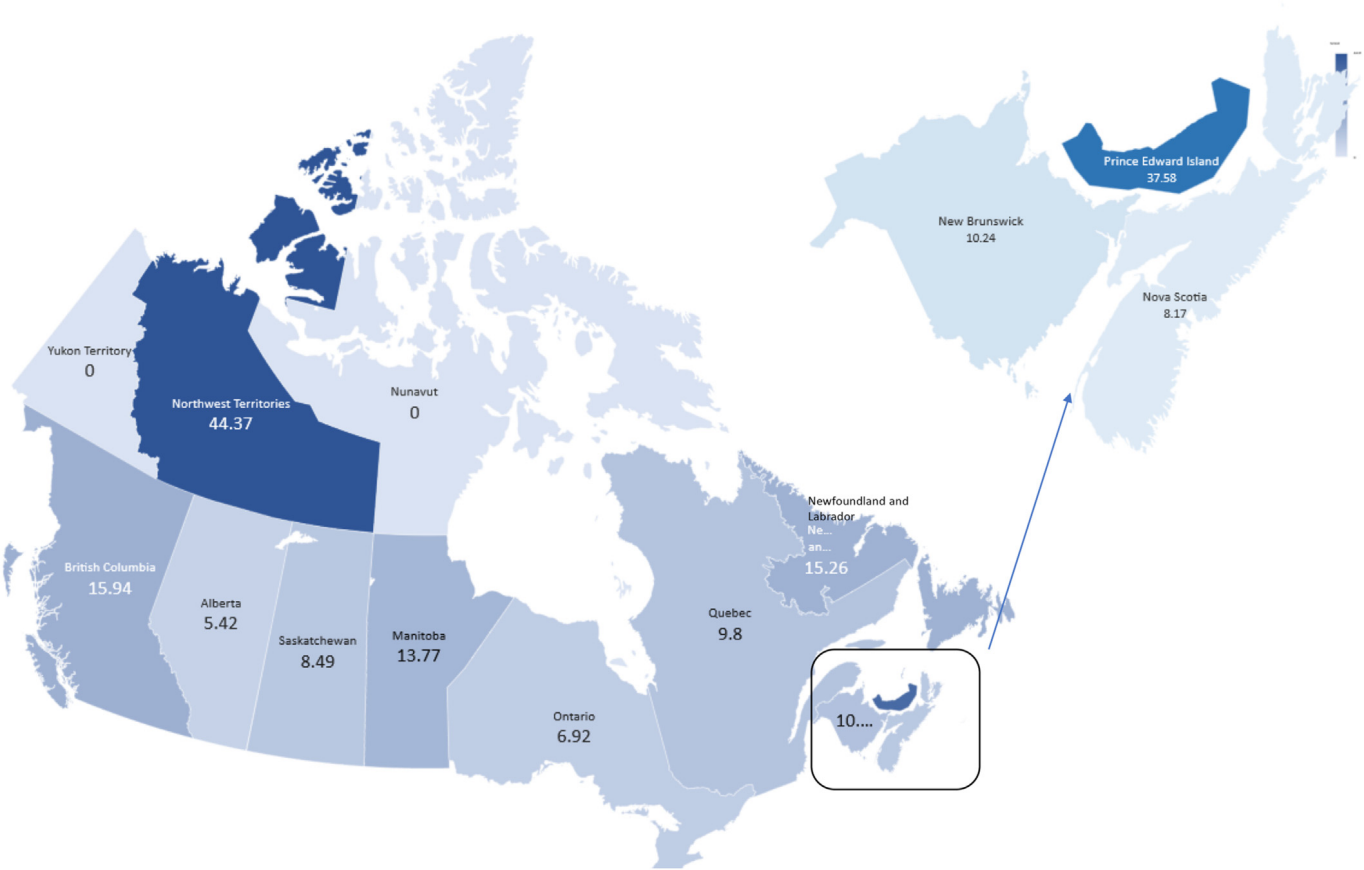
A map of Canada and proportion of responses received per million population in each province/territory to account for variable population densities.

We obtained 353 complete responses. Except for the Canadian territories of Yukon and Nunavut, at least 5 responses per million population were obtained from each province, indicating adequate representation from regions with different population densities. This included the 3 most densely populated provinces of British Columbia (15.9), Ontario (6.9) and Québec (9.8) (Figure 2). Of all participants, 35.1% were from low-performing provinces and 29.2% were physicians. Most respondents identified themselves as female (75.6%) and White (73.9%) (Table 1).

### Most Important Barriers

#### Role Perception and Multidisciplinary Involvement

Poor role perception and lack of multidisciplinary involvement emerged as barriers. Whereas 59.2% stated that discussion about living donation is a part of their role, about half of the participants thought that the transplant team is best suited to discuss LDKT (52.7%) or felt that the entire multidisciplinary team was engaged in promoting LDKT (46.9%). Also, only 51.8% initiated discussions about LDKT with patients. More importantly, though the odds of agreeing that discussions about living donation are a part of their current role did not differ (aOR = 0.72, 95% CI:0.43–1.19), HPs from low-performing provinces had lower odds of initiating discussions about LDKT with their patients (aOR = 0.29, 95% CI: 0.16–0.52) and higher odds of agreeing that the transplant team is best suited to discuss LDKT (aOR = 2.55, 95% CI: 1.57–4.15) than HPs from high-performing provinces (Table 2).

**Table 2:** Identifying and comparing barriers to LDKT by LDKT performance^a^.

Theme	% of respondents who agreed/strongly agreed	Univariate analysis	Multivariate analysis^b^
		Ref: high-performing provinces	Ref: high-performing provinces
Theme 1: Communication			
• There is good communication between the transplant center and referring centers^c^	36.8	_0.99_1.53_2.38_	_0.99_1.73_3.01_
• The donor evaluation team and recipient evaluation team do not communicate well with each other^c^	26.6	_0.30_0.57_1.08_	_0.26_0.51_1.01_
Theme 2: Referral process			
• I am aware of how to refer living donors to transplant centers	66.9	_0.63_1.01_1.60_	_0.46_0.79_1.35_
• The referral and evaluation processes for LDKT is very disorganized^c^	26.5	_0.65_1.11_1.90_	_0.61_1.09_1.92_
Theme 3: Role perception and multidisciplinary involvement			
• Discussions about living donation is a part of my current role	59.2	_0.63_0.98_1.53_	_0.43_0.72_1.19_
• The transplant team is best suited to discuss LDKT	52.7	_1.34_2.11_3.31_^d^	_1.57_2.55_4.15_^d^
• We engage the entire multidisciplinary team in promoting LDKT^c^	46.9	_0.45_0.73_1.19_	_0.35_0.61_1.06_
• I initiate discussions about LDKT with my patient	51.8	_0.41_0.63_0.98_^d^	_0.16_0.29_0.52_^d^
Theme 4: HP’s education, training and comfort			
• LDKT was a part of my training	43.9	_0.60_0.93_1.44_	_0.43_0.71_1.19_
• I feel comfortable counseling patients with kidney failure on LDKT	56.9	_0.59_0.92_1.43_	_0.39_0.66_1.12_
• I feel comfortable discussing kidney donation with a suitable donor	47.3	_0.93_1.44_2.24_	_0.83_1.34_2.18_
• I am aware of the major risks of living donation to the donor	54.7	_0.97_1.52_2.36_	_0.81_1.37_2.30_
Theme 5: HPs perception on LDKT			
• LDKT is the gold standard of care for patients with kidney failure	83.0	_0.72_1.32_2.41_	_0.57_1.10_2.15_
• I know of healthcare providers who feel negatively toward LDKT^c^	9.4	_0.41_0.92_2.06_	_0.59_1.40_3.35_
Theme 6: Patient-level barriers as defined by HP			
• LDKT should only be discussed with those who are likely to move forward with it	13.3	_0.62_1.17_2.21_	_0.94_1.92_3.93_
• There are patient-level factors that prevent discussions related to LDKT^c^	44.8	_0.63_1.02_1.66_	_0.52_0.88_1.49_
Theme 7: Resources and infrastructure			
• My province actively promotes LDKT^c^	60.9	_0.18_0.29_0.48_^d^	_0.16_0.27_0.47_^d^
• The current system does not facilitate the evaluation of donors^c^	28.8	_0.87_1.48_2.5_	_0.81_1.41_2.47_
• There are specific people hired to help patients with LDKT^c^	44.2	_0.91_1.49_2.46_	_0.75_1.28_2.18_
• If I had more resources, I would discuss LDKT more with my patients	48.7	_0.99_1.53_2.38_	_1.31_2.13_3.47_^d^

HP, health professionals; LDKT, living donor kidney transplantation.

Participants were asked to rate statements on a Likert scale and percentage who agreed or strongly agreed are reported. We then conducted a logistic regression of the level of agreement with these statement (agreement vs. disagreement/neutral) by LDKT performance in a univariate and a multivariate framework.

a High-performing provinces were those with living donor rates consistently above the national average of 15 living donors per million population (British Columbia, Ontario, Alberta, Manitoba, and North-West Territories) whereas low-performing provinces were those whose rates were consistently below (Québec, Nova Scotia, New Brunswick, Saskatchewan, Newfoundland and Labrador, and Prince Edward Island).

b Adjusted for the following respondent characteristics: gender they identified with, their role in the multidisciplinary team, self reported race and years of experience.

c The option do not know/not applicable was possible.

d Significant findings.

#### Resources and Infrastructure

Lack of resources and infrastructure also emerged as barriers. Not all agreed that their province actively promoted LDKT (60.9%) and about 28.8% felt that the current system does not facilitate the evaluation of donors. Whereas 44.2% agreed that there are specific people hired to help patients with LDKT and about 48.7% stated that if they had more resources, they would discuss LDKT more with patients. Notably, HPs from low-performing provinces had lower odds of agreeing that their province actively promoted LDKT (aOR = 0.27, 95% CI: 0.16–0.48), and higher odds of agreeing that if they had more resources, they would discuss LDKT more with patients (aOR = 2.06, 95% CI: 1.25–3.40) (Table 2).

#### HP’s Education, Training and Comfort

LDKT was a part of the training of only 43.9% of HPs. Thus, only 56.9% felt comfortable counseling patients with kidney failure on LDKT, 47.3% felt comfortable discussing kidney donation with a suitable donor and 54.7% were aware of the major risks of living donation to the donor. The level of agreement did not vary by performance (Table 2).

#### Patient as the Barrier

Although the level of agreement did not vary by performance, 44.8% did agree that patient-level factors prevented discussions related to LDKT and 13.3% agreed that LDKT should only be discussed with those who are likely to move forward with it (Table 2).

#### Other Barriers

Poor communication between transplant and referring centers emerged as a barrier because only 36.8% agreed that there is good communication. However, only 26.6% agreed that the donor and recipient teams do not communicate well with each other. Also, the level of agreement did not vary by LDKT performance. The referral process and poor perception about LDKT emerged as minor barriers because the majority agreed that LDKT is the gold standard of care for patients with kidney failure (83%) and only 9.4% knew of other HPs who feel negatively toward LDKT. Also, the level of agreement did not vary by performance (Table 2).

#### Comparing Barriers by HP Characteristics

The 2 most important HP characteristics that were associated with the level of agreement were the role of the HP and years of experience (Table 3). Physicians had 2 to 8 times higher odds of agreeing with several statements, including LDKT being the gold standard of care for patients with kidney failure (aOR = 8.48, 95% CI: 2.88–24.97) than nonphysicians. More years of experience was also associated with lower perception of barriers identified, such as >10 years of experience was associated with lower odds of agreeing that patient-level factors prevent discussions related to LDKT (aOR = 0.51, 95% CI: 0.31–0.85). When compared with male gender, female gender was overall not associated with the level of agreement except for 2 statements that pertained to resources (aOR = 0.37, 95 CI%: 0.19–0.72) and engaging the entire multidisciplinary team in promoting LDKT (aOR = 0.37, 95% CI: 0.18–0.79). The race of the HP was associated with the level of agreement of some statements. When compared with White HPs, non-White HPs had higher odds of agreeing that the transplant team is best suited to discuss LDKT (aOR = 1.92, 95% CI: 1.07–3.45); knowing health care providers who feel negatively toward LDKT (aOR = 3.92, 95% CI: 1.56–9.85); agreeing that LDKT should only be discussed with those who are likely to move forward with it (aOR = 5.00, 95% CI: 2.39–10.46), and; agreeing that more resources would increase LDKT discussions with patients (aOR = 2.22, 95% CI: 1.22–4.04) (Table 3).

**Table 3:** Comparing barriers to LDKT by characteristics of the HP^b^.

Theme	Role: physician (Ref: nonphysician)	Yr of experience: >10 yr (Ref: ≤10 yr)	Gender: female (Ref: male)	Race: non-White (Ref: White)
Theme 1: Communication				
• There is good communication between the transplant center and referring centers^a^	_1.11_2.11_4.00_^c^	_0.81_1.36_2.29_	_0.35_0.73_1.51_	_0.57_1.09_2.10_
• The donor evaluation team and recipient evaluation team do not communicate well with each other^a^	_0.56_1.13_2.28_	_0.61_1.15_2.14_	_0.61_1.31_2.82_	_0.39_0.86_1.88_
Theme 2: Referral process				
• I am aware of how to refer living donors to transplant centers	_2.74_5.80_12.28_^c^	_1.70_2.78_4.54_^c^	_0.73_1.64_3.69_	_0.45_0.86_1.66_
• The referral and evaluation processes for LDKT is very disorganized^a^	_0.57_1.10_2.13_	_0.98_1.74_3.08_	_0.69_1.44_2.98_	_0.44_0.91_1.88_
Theme 3: Role perception and multidisciplinary involvement				
• Discussions about living donation is a part of my current role	_2.96_5.79_11.30_^c^	_0.84_1.33_2.12_	_0.54_1.10_2.26_	_0.36_0.66_1.22_
• The transplant team is best suited to discuss LDKT	_0.37_0.64_1.12_	_0.42_0.65_1.02_	_0.46_1.10_1.64_	_1.07_1.92_3.45_^c^
• We engage the entire multidisciplinary team in promoting LDKT^a^	_1.59_3.06_5.89_^c^	_1.28_2.14_3.56_^c^	_0.18_0.37_0.79_^c^	_0.46_0.88_1.69_
• I initiate discussions about LDKT with my patient	_9.20_20.62_46.23_^c^	_0.85_01.40_2.31_	_0.35_0.80_1.79_	_0.27_0.52_1.01_
Theme 4: HP’s education, training and comfort				
• LDKT was a part of my training	_2.91_5.24_9.44_^c^	_0.60_0.95_1.51_	_0.55_1.06_2.05_	_0.68_1.24_2.26_
• I feel comfortable counseling patients with kidney failure on LDKT	_3.91_7.88_15.90_^c^	_1.48_2.41_3.91_^c^	_0.73_1.57_3.36_	_0.42_0.81_1.54_
• I feel comfortable discussing kidney donation with a suitable donor	_1.43_2.49_4.33_^c^	_1.07_1.69_2.66_^c^	_0.84_1.60_3.03_	_0.64_1.15_2.07_
• I am aware of the major risks of living donation to the donor	_2.48_4.68_8.83_^c^	_2.02_3.30_5.39_^c^	_0.92_1.91_3.96_	_0.50_0.94_1.78_
Theme 5: HPs perception on LDKT				
• LDKT is the gold standard of care for patients with kidney failure	_2.88_8.48_24.97_^c^	_1.34_2.42_4.37_^c^	_0.18_0.44_1.09_	_0.32_0.68_1.42_
• I know of health care providers who feel negatively toward LDKT^a^	_0.19_0.55_1.61_	_1.16_3.02_7.84_^c^	_0.21_0.71_2.43_	_1.56_3.92_9.85_^c^
Theme 6: Patient-level barriers as defined by HP				
• LDKT should only be discussed with those who are likely to move forward with it	_0.27_0.62_1.42_	_0.49_0.95_1.81_	_0.40_0.98_2.40_	_2.39_5.00_10.46_^c^
• There are patient-level factors that prevent discussions related to LDKT^a^	_0.65_1.18_2.15_	_0.31_0.51_0.85_^c^	_0.98_1.93_3.81_	_0.67_1.25_2.34_
Theme 7: Resources and infrastructure				
• My province actively promotes LDKT^a^	_0.73_1.38_2.63_	_0.71_1.20_2.01_	_0.34_0.70_1.43_	_0.45_0.89_1.76_
• The current system does not facilitate the evaluation of donors^a^	_0.76_1.43_2.71_	_0.69_1.22_2.14_	_0.46_0.95_1.93_	_0.57_1.14_2.28_
• There are specific people hired to help patients with LDKT^a^	_1.12_2.11_3.97_^c^	_0.56_0.94_1.58_	_0.30_0.61_1.23_	_0.40_0.79_1.56_
• If I had more resources, I would discuss LDKT more with my patients	_0.34_0.60_1.05_	_0.61_0.95_1.48_	_0.19_0.37_0.72_^c^	_1.22_2.22_4.04_^c^

HP, health professional; LDKT, living donor kidney transplantation.

Participants were asked to rate statements on a Likert scale and we then conducted a logistic regression of the level of agreement with these statement (agreement vs. disagreement/neutral) by HP’s characteristics in a multivariate framework.

a The option do not know/not applicable was possible.

b Ajusted for LDKT performance, gender they identified with, their role in the multidisciplinary team, self reported race and years of experience.

c Significant findings.

#### HP-identified Priorities to Increase LDKT

Overall priorities to increase LDKT are presented in Table 4. In high-performing provinces, the following 3 statements were ranked as top priorities to increase LDKT: (i) create standardized referral and evaluation guidelines; (ii) organize and streamline the evaluation of living donors and their recipients better; and (iii) provide more education and training to HPs. In low-performing provinces, the top 3 priorities were as follows: (i) organize and streamline the evaluation of living donors and their recipients better; (ii) improve communication between the referring team and the transplant team; and (iii) provide more education to the recipients and donor candidates. More funding to support resources and other personnel were ranked lower. The top 3 priorities in each province are presented in Supplementary Table S1, descriptive comments were analyzed and additional themes generated are presented as other priorities in Table 4. These themes pertained to public and patient education and financial assistance for donors and recipients.

**Table 4:** Health professional ranked priorities to increase living donor kidney transplantation

Overall ranking of statements (% of participants who picked these statements in their top 3 choices)
1. Create standardized referral and evaluation guidelines (56.4%)
2. Organize and streamline evaluation of living donors and their recipients better (52.7%)
3. Provide more education and training to health professionals (44.8%)
4. Provide more education to the recipients and donor candidates (42.3%)
5. Improve communication between the referring team and the transplant team (39.1%)
6. Engage every team member in discussions about living donation (23.5%)
7. More funding to support resources and other personnel (28.9%)
8. Improve general attitudes among health professionals toward living donation (8.5%)
9. Other (5.1%)
Other emerging themes based on the review of the descriptive comments (in no particular order)
• Increasing public education
• Improved peer support
• Financial support for the donor and attaining financial neutrality

## Discussion

In this national survey of over 350 HPs, we identified several system-level barriers to LDKT and report that some were more prevalent in low-performing provinces. Poor communication between treating teams, and HPs lack of education, training and comfort emerged as barriers to LDKT, and the level of agreement did not vary by performance. Nevertheless, barriers related to resources, LDKT promotion and role perception were significantly more prominent in low-performing provinces of Canada. HPs from low-performing provinces had 73% lower odds of stating that their province actively promoted LDKT, had 71% lower odds of initiating discussions about LDKT with their patients, had 2.1 times higher odds of stating that if they had more resources, they would discuss LDKT more with patients, and had 2.6 times higher odds of stating that the transplant team is best suited to discuss LDKT. We also report that certain HP characteristics, in particular, less than 10 years of experience and nonphysician role, are significantly associated with a higher perception of barriers. HPs identified the need to improve several system-level inefficiencies such as better communication between treating teams, and streamlining the donor and recipient evaluation, as top priorities to improve LDKT over patient education. To our knowledge, this is the first study that has attempted to identify system-level factors that drive differences in LDKT performance in a real-world setting.

Previous efforts to understand system-level barriers to LDKT have generally focused on how system-level factors are contributing to disparities in access to LDKT,^37^^,^^38^ or have been patient led-efforts to understand system-level barriers to LDKT.^14^^,^^20^ Patient perspective is an important tool toward facilitating patient-centered care^39^; however, HPs also provide key understanding of system-level deficiencies and inefficiencies.^40^ The perspective of HPs about the care processes they operate within has been proposed to be an important dimension to improve the care delivered to patients.^41^^,^^42^ Multiple consensus guidelines and recommendations have been published to improve LDKT rates and address barriers to LDKT.^2^^,^^3^^,^^10^, ^11^, ^12^ These guidelines have emphasized educating general practitioners, primary nephrologists and dialysis staff so that patients have access to transplant education during earlier stages of the disease.^10^^,^^43^ In the developing world where 85% to 100% of transplants are from living donors, many have identified HP’s lack of awareness of LDKT as a barrier to implementing it.^44^, ^45^, ^46^ In the UK, 3 models of which one entails HP’s contacting the patients’ families with information about kidney donation are being explored to develop a multicomponent intervention to increase LDKT.^47^ Thus, our work complements current efforts and agendas and has practice and policy implications.

HPs from low-performing provinces were significantly less likely to initiate discussions about LDKT and felt the transplant team is best suited to discuss LDKT. This is a problematic finding because early discussions with patients are essential to the pursuit of LDKT.^11^^,^^43^^,^^48^ Delaying these discussions until the patient is seen by the transplant center decreases the chances of patients receiving LDKT and maybe a big contributor to low LDKT performance and lead to less pre-emptive transplantations. Another problematic finding is that nonphysicians seem to experience more barriers than physicians. The crucial role that nonphysicians, such as social workers, dialysis nurses, and chronic kidney disease nurses, play in a patient’s decision to pursue transplantation is well described.^2^^,^^49^, ^50^, ^51^, ^52^, ^53^ Nurses and nurse educators often spend more time with patients than physicians, especially with patients who are on dialysis. Thus, improving their engagement, education, and role perception may be one of the strongest modifiable factors to increasing LDKT that we have identified. HPs may have insufficient time or training to administer transplant education and may not have access to transplant education materials.^54^^,^^55^ Our recent work has expanded on how the province of British Columbia has achieved this by creating a commitment to collaboration and cultivating distributed expertise.^15^ Working groups, committees and cross-provincial events in the province have created strong communities of practice and conveyed an awareness of and knowledge about LDKT. Collaborative networks have helped the development and circulation of educational resources about LDKT for not just patients and donors, but also HPs. These efforts are likely driving high performance in this province.

Some other findings merit discussion. Surprisingly more funding to support resources and other personnel were not ranked very highly by our participants as strategies to improve LDKT. This indicates that current barriers to LDKT are at the level of implementation as pointed by 1 participant, “more funding is always helpful, however, it needs to be used appropriately.” We have explored the role of an activity-based funding model that was implemented in British Columbia as a facilitator to LDKT.^15^ However, this model was implemented after an economic analysis in the province and may require a similar analysis in every transplant jurisdiction. Also, several responses varied by the race and gender of the HP. HP’s gender, years of experience, race and perceived role are all factors that are associated with care process, outcomes, and other measures of health care delivery.^31^, ^32^, ^33^, ^34^, ^35^, ^36^ Female HPs engage in more communication that can be considered patient-centered and have longer visits than male HPs.^34^ This may be why they were less likely to agree that more resources would lead them to discuss LDKT more with their patients. However, not all findings can be explained by conventional wisdom and require further exploration.

Our study has several strengths. This national study was conducted in a setting that offers real-world disparity in LDKT performance and has clinical and policy implications. Population characteristics do not explain these differences. For example, in 2017, 7.3% of Canadians aged 12 and older had diabetes.^56^ Ontario, a high-performing province, and New Brunswick, a low-performing province, had a diabetes prevalence of 8.0% and 9.5%, respectively, which is higher than the national average. British Columbia, a high-performing province, and Québec, a low-performing province, had a diabetes prevalence of 5.9% and 6.6% which is lower than the national average.^56^ Similar statistics exist for hypertension as well.^57^ Thus, the prevalence of common medical comorbidities that preclude donation do not explain differences in LDKT. We used the Canadian health care system as a model; however, our findings are applicable to most countries because the referral process of transplantation is essentially the same. Our findings are of even more relevance in countries where living donation is the only or main option for patients with kidney failure. We used several best practices in survey design and administration to ensure our methods were robust. Our interdisciplinary team with expertise in qualitative and quantitative methods meticulously developed statements in the survey. We were able to recruit not just academic HPs but also those practicing in the community and had good representation from nonphysicians to obtain diverse viewpoints.

We, however, acknowledge the following limitations. We could not obtain equal representation from all Canadian provinces, but our sample size was large enough to enable comparative analysis and draw plausible conclusions. The non-White category was a convenience sample, and we acknowledge that this may not capture the nuances of HP behaviors and their inherent biases. As with any survey study, our findings are at risk of subjective and social acceptability bias despite us adopting several good-practice recommendations in survey design and administration. The COVID-19 pandemic could have influenced some responses. We invited the pilot participants to take our survey again and this may have introduced bias in our analysis. Although, even if all 16 pilot participants retook the survey given the large sample size, we anticipate this bias to be minimal. We were unable to calculate a precise response rate, and this is a methodological limitation; however, as depicted in the study flow diagram our response rate was likely in the 25% to 30% range. HP surveys are at risk of low response rates and responses as low as 10% have been reported.^58^, ^59^, ^60^ Our response rate is comparable to several other surveys involving HPs. We also attempted to address the risk of nonresponse bias by having a dual recruitment strategy whereby we compiled a list of dialysis centers and kidney care clinics in Canada and contacted their managers or directors directly. This is a unique target population because many are not affiliated with academic societies, yet they play a crucial role in the daily clinical management of patients. We believe this to be a potential strategy to have addressed the risk of nonresponse bias.

Despite this, our findings are extremely important because LDKT is the best treatment option for patients with kidney failure and there is limited understanding of system-level barriers to LDKT. We compared the magnitude of these barriers by LDKT performance and HP characteristics and identified key differences. Observed differences provide empirical evidence to support early patient discussions about LDKT, educating HPs and implementing system-level changes to address inefficiencies in the evaluation process. Most importantly, we have identified some modifiable factors to increasing LDKT which can inform interventional work. This includes increasing the engagement of and role perception of nonphysicians, improving HP’s education, training and comfort, and providing them with more resources to discuss LDKT with patients. This can serve as a roadmap for any transplant program seeking to undertake a system-level approach to increasing LDKT and has international implications.

In conclusion, poor communication between treating teams, poor role perception and HPs lack of education, training and comfort emerged as system-level barriers to LDKT and even HPs themselves identified addressing system-level inefficiencies as priorities to increase LDKT over patient education. More importantly, we report that poor role perception, low resources, and poor infrastructure may be driving differences in LDKT performance in a real-world setting. Our findings have policy implications and can build on the current patient-level work that others are pursuing to increasing LDKT. We recommend that interventions specifically designed to eliminate these system-level barriers should be implemented in conjunction with patient-level interventions as part of the “LDKT first” strategy for the treatment of end-stage kidney disease.

## Disclosure

SS has received an education grant from Amgen Canada to increase living donor kidney transplantation and improve rekidney transplantation. AA receives honoraria as a consultant/advisory board from Otsuka, Janssen, Astrazeneca, Bayer, and Royal College International (Canada). He also has grants or clinical trials with Otsuka and Janssen. RSS has participated in advisory boards for Amgen, Otsuka, and Astra-Zeneca. All the other authors declared no competing interests.

## Supplementary Material

Supplementary File (PDF)

**Table S1.** The top 3 ranked priorities to increase living donor kidney transplantation by province

**Survey.**

**STROBE Statement.**
