# Supplementary material for: Identifying Modifiable System-Level Barriers to Living Donor Kidney Transplantation
Source: Kidney Int Rep. 2022 Sep 9;7(11):2410–20. doi: 10.1016/j.ekir.2022.08.028 (PMC9751709; doi:10.1016/j.ekir.2022.08.028)
Supplement: Supplementary File (PDF) [file mmc1.pdf]

Supplement 1: Modified STROBE Statement—checklist of items that should be included in reports of observational studies (Cohort/Cross-sectional and case-control studies)

	Item No	Recommendation	
**Title and abstract**	1	(*a*) Indicate the study's design with a commonly used term in the title or the abstract	Yes
		(*b*) Provide in the abstract an informative and balanced summary of what was done and what was found	Yes
**Introduction**			
Background/rationale	2	Explain the scientific background and rationale for the investigation being reported	4-5
Objectives	3	State specific objectives, including any prespecified hypotheses	5
**Methods**			
Study design	4	Present key elements of study design early in the paper	5-6
Setting	5	Describe the setting, locations, and relevant dates, including periods of recruitment, exposure, follow-up, and data collection	6-7
Participants	6	(*a*) *Cohort study*—Give the eligibility criteria, and the sources and methods of selection of participants. Describe methods of follow-up<br><br>*Case-control study*—Give the eligibility criteria, and the sources and methods of case ascertainment and control selection. Give the rationale for the choice of cases and controls<br><br>*Cross-sectional study*—Give the eligibility criteria, and the sources and methods of selection of participants	5
Variables	7	Clearly define all outcomes, exposures, predictors, potential confounders, and	7

		effect modifiers. Give diagnostic criteria, if applicable	
Data sources/ measurement	8*	For each variable of interest, give sources of data and details of methods of assessment (measurement).	*Attached as supplement*
Bias	9	Describe any efforts to address potential sources of bias	6-7
Study size	10	Explain how the study size was arrived at (if applicable)	Study flow diagram
Quantitative variables	11	Explain how quantitative variables were handled in the analyses. If applicable, describe which groupings were chosen and why	8
Statistical methods	12	(*a*) Describe all statistical methods, including those used to control for confounding	8
		(*b*) Describe any methods used to examine subgroups and interactions	8
		(*c*) Explain how missing data were addressed	NA
		(*d*) *Cohort study*—If applicable, explain how loss to follow-up was addressed<br><br>*Case-control study*—If applicable, explain how matching of cases and controls was addressed<br><br>*Cross-sectional study*—If applicable, describe analytical methods taking account of sampling strategy	8
		(*e*) Describe any sensitivity analyses	NA
**Results**			
Participants	13*	(a) Report numbers of individuals at each stage of study—eg numbers potentially eligible, examined for eligibility, confirmed eligible, included in the study, completing follow-up, and analyzed	8-9

		(c) **Use of a flow diagram**	Figure 1
Descriptive data	14*	(a) Give characteristics of study participants (eg demographic, clinical, social) and information on exposures and potential confounders	Table 1
		(b) Indicate number of participants with missing data for each variable of interest	NA
		(c) *Cohort study*—Summarise follow-up time (eg, average and total amount)	
Outcome data	15*	*Cohort study*—Report numbers of outcome events or summary measures over time	
		Case-control study—Report numbers in each exposure category, or summary measures of exposure	
		Cross-sectional study—Report numbers of outcome events or summary measures	*Table 1*
Main results	16	(*a*) Give unadjusted estimates and, if applicable, confounder-adjusted estimates and their precision (eg, 95% confidence interval). Make clear which confounders were adjusted for and why they were included	9-10
Other analyses	17	Report other analyses done—eg analyses of subgroups and interactions, and sensitivity analyses	10-11
**Discussion**			
Key results	18	Summarise key results with reference to study objectives	11-12
Limitations	19	Discuss limitations of the study, taking into account sources of potential bias or imprecision. Discuss both direction and magnitude of any potential bias	15-16
Interpretation	20	Give a cautious overall interpretation of results considering objectives, limitations, multiplicity of analyses, results from	16-17

		similar studies, and other relevant evidence	
Generalisability	21	Discuss the generalisability (external validity) of the study results	14-15

*Give information separately for cases and controls in case-control studies and, if applicable, for exposed and unexposed groups in cohort and cross-sectional studies.

Note: An Explanation and Elaboration article discusses each checklist item and gives methodological background and published examples of transparent reporting. The STROBE checklist is best used in conjunction with this article (freely available on the Web sites of PLoS Medicine at http://www.plosmedicine.org/, Annals of Internal Medicine at http://www.annals.org/, and Epidemiology at http://www.epidem.com/). Information on the STROBE Initiative is available at www.strobe-statement.org.

Supplement 2 :
Supplementary Table 1: The top three ranked priorities to increase living donor kidney transplantation by province

Alberta (n=24)<br><br>1. Create standardized referral and evaluation guidelines<br>2. Organize and streamline evaluation of living donors and their recipients better<br>3. Provide more education to the recipients and donor candidates
British Columbia (n=82)<br><br>1. Organize and streamline evaluation of living donors and their recipients better<br>2. Improve communication between the referring team and the transplant team<br>3. Provide more education to the recipients and donor candidates
Manitoba (n=19)<br><br>1. Create standardized referral and evaluation guidelines<br>2. Organize and streamline evaluation of living donors and their recipients better<br>3. Provide more education and training to health professionals
New Brunswick (n=8)<br><br>1. Create standardized referral and evaluation guidelines<br>2. Provide more education to the recipients and donor candidates<br>3. Provide more education and training to health professionals
Newfoundland and Labrador (n=8)<br><br>1. Provide more education and training to health professionals<br>2. Create standardized referral and evaluation guidelines<br>3. Provide more education to the recipients and donor candidates
North-West Territories (n=2)<br><br>1. Provide more education and training to health professionals<br>2. Organize and streamline evaluation of living donors and their recipients better<br>3. Provide more education to the recipients and donor candidates
Nova Scotia (n=8)

1. Organize and streamline evaluation of living donors and their recipients better<br>2. Provide more education to the recipients and donor candidates<br>3. Create standardized referral and evaluation guidelines
Ontario (n=102)<br><br>1. Create standardized referral and evaluation guidelines<br>2. Organize and streamline evaluation of living donors and their recipients better<br>3. Provide more education and training to health professionals
Prince Edward Island (n=6)<br><br>1. Organize and streamline evaluation of living donors and their recipients better<br>2. Provide more education and training to health professionals<br>3. Create standardized referral and evaluation guidelines
Quebec (n=84)<br><br>1. Create standardized referral and evaluation guidelines<br>2. Provide more education and training to health professionals<br>3. Organize and streamline evaluation of living donors and their recipients better
Saskatchewan (n=10)<br><br>1. Improve communication between the referring team and the transplant team<br>2. Provide more education and training to health professionals<br>3. Organize and streamline evaluation of living donors and their recipients better

Supplement 3 : Survey

Health Professional Identified Barriers to Living Donor Kidney Transplantation

Dear Colleagues,

Thank you for agreeing to participate in our research study supported by the Canadian Donation and Transplantation Research Program. We want to learn more about the barriers to living donor kidney transplantation that health professionals, **like you,** are facing across different provinces. Participating in this study will inform us on how to improve the services provided to you and your patients and obtain funding to do so. Upon completion of the survey, you may submit your name to be entered in a raffle to receive one of three $100 gift certificates as a token of our appreciation.

The survey will take approximately 10 minutes to complete and there are no anticipated risks. Please be assured that the survey responses will be de-identified and only a collective analysis will be conducted. Survey data will be available only to the investigators in this study and will only be used for the purpose of this study.

By completing this survey, you are consenting to participate in the research study. Please note that this survey should only be completed once. Your participation is voluntary, and you may choose to withdraw your participation at any time. For any questions or concerns, our contact information is below.

Sincerely,

Principal Investigator: Shaifali Sandal[1]

Co-Investigators and Collaborators: Nandini Dendukuri[1], David Landsberg[2], Marcelo Cantarovich[1], Catherine Weber[1], Ahsan Alam[1,] Julio F Fiore Jr[1], Prosanto Chaudhury[1], Marie-Chantal Fortin[3]

[1]McGill University, Montreal, QC; [2]University of British Columbia, Vancouver, BC;

[3]Centre Hospitalier de l’Université de Montréal, Montreal, QC

Contact information of investigators: Royal Victoria Hospital Glen Site, D05-7160, 1001 boul Decarie, Montreal, QC, Canada H4A 3J1. Tel: +1 (514) 934-1934 ext. 35203. Fax: +1 (514) 938-7050. Email: shaifali.sandal@mcgill.ca

Contact information for the Office of the Ombudsman: 1650 Cedar Room E6.164, Montreal, QC, Canada, H3G 1A4. Tel: +1 (514) 934-1934 ext 48306. Email: ombudsman@muhc.mcgill.ca

1. What **best** describes your primary occupation role?
    - Administrative assistant
    - Dietician
    - Nephrologist (donor)
    - Nephrologist (referring)
    - Nephrologist (transplant)
    - Nurse (chronic kidney disease)
    - Nurse (dialysis)
    - Nurse (manager)
    - Transplant coordinator
    - Patient partner
    - Social worker
    - Technician (dialysis)
    - Other (please specify) ______________

2. How long have you been working in the field of nephrology or transplantation?
    - <1 year
    - 1-5 years
    - 6-10 years
    - 11-20 years
    - >20 years

3. In what province or territory do you currently practice?
    - Alberta
    - British Columbia
    - Manitoba
    - New Brunswick
    - Newfoundland and Labrador
    - North-West Territories
    - Nova Scotia

- Nunavut
- Ontario
- Prince Edward Island
- Québec
- Saskatchewan
- Yukon

4. The following questions pertain to your personal thoughts/opinions/experiences related to living donor kidney transplantation (LDKT) even if it is not a part of your current job. On a scale of 1-5 (1 being strongly disagree and 5 being strongly agree), please rate the following statements.

	1 (Strongly disagree)	2	3	4	5 (Strongly agree)
Discussions about living donation is a part of my current role					
LDKT was a part of my training					
I feel comfortable counselling patients with kidney failure on LDKT					
LDKT is the gold standard of care for patients with kidney failure					
I feel comfortable discussing kidney donation with a suitable donor					
If I had more **resources**, I would discuss LDKT more with my patients					
I am aware of the major risks of living donation to the donor					

LDKT should only be discussed with those who are likely to move forward with it					
I initiate discussions about LDKT with my patient					
The transplant team is best suited to discuss LDKT					
I am aware of how to refer living donors to transplant centres					

5. On a scale of 1-5 (1 being strongly disagree and 5 being strongly agree), please rate the following statements on living donor kidney transplantation (LDKT) with respect to your organization/centre/hospital/clinic. **We ask that you answer these questions based on your experiences prior to the COVID-19 pandemic**

	1 (Strongly disagree)	2	3	4	5 (Strongly agree)	Do not know/Not applicable
The referral and evaluation processes for LDKT is very disorganized						
The current system does not facilitate the evaluation of donors						
There is good communication between the transplant center and referring centers						
The donor evaluation team and recipient evaluation team						

do not communicate well with each other						
We engage the entire multi-disciplinary team in promoting LDKT						
My province actively promotes LDKT						
There are patient-level factors that prevent discussions related to LDKT						
I know of healthcare providers who feel negatively towards LDKT						
There are specific people hired to help patients with LDKT						

6. Our research has identified the following approaches that should be taken to increase LDKT. Please rank them by priority, 1 being the most important and 9 being the least.

- Improve communication between the referring team and the transplant team
- Organize and streamline evaluation of living donors and their recipients better
- Create standardized referral and evaluation guidelines
- Provide more education and training to health professionals
- Provide more education to the recipients and donor candidates
- Engage every team member in discussions about living donation
- Improve general attitudes amongst health professionals towards living donation
- More funding to support resources and other personnel
- Other, please type

____

------- Next Page -------

We will now ask you some demographical questions: (All optional)

7. What is your age?

a. <25 years old
b. 25-34 years old
c. 35-44 years old
d. 45-54 years old
e. 55-64 years old
f. ≥65 years old
g. Prefer not to answer

8. What gender do you identify as?

a. Male
b. Female
c. Prefer not to answer
d. Other (please specify) ___________________

9. What is your ethnicity or race? (Check all that apply)

e. Asian
f. Black or African American
g. Caucasian
h. Hispanic or Latin
i. Indigenous
j. Middle Eastern or North African
k. Pacific Islander
l. South Asian
m. Prefer not to answer
n. Other (please specify) ___________________

Final comments:

10. We tried to make this survey as comprehensive as possible, but you may feel that there are topics that we have not covered. If you have any additional comments, please elaborate below using as much space as needed.

------- Next Page -------

Thank you for completing the survey. Please enter your email address below so that we can enter your name in our raffle.
